# Dispersion of Carbon Nanotubes with “Green” Detergents

**DOI:** 10.3390/molecules26102908

**Published:** 2021-05-14

**Authors:** Kazuo Umemura, Ryo Hamano, Hiroaki Komatsu, Takashi Ikuno, Eko Siswoyo

**Affiliations:** 1 Department of Physics, Faculty of Science Division II, Tokyo University of Science, 1-3 Kagurazaka, Shinjuku 1628601, Japan; r.hamano.tus@gmail.com; 2 Department of Applied Electronics, Faculty of Advanced Engineering, Tokyo University of Science, 6-3-1 Niijuku, Katsushika, Tokyo 1258585, Japan; meicun2006@163.com (H.K.); tikuno@rs.tus.ac.jp (T.I.); 3 Department of Environmental Engineering, Islamic University of Indonesia (UII), Jl. Kaliurang Km 14.5, Yogyakarta 55584, Indonesia; eko_siswoyo@uii.ac.id

**Keywords:** carbon nanotube, green molecules, natural detergents, coconuts, bamboo

## Abstract

Solubilization of carbon nanotubes (CNTs) is a fundamental technique for the use of CNTs and their conjugates as nanodevices and nanobiodevices. In this work, we demonstrate the preparation of CNT suspensions with “green” detergents made from coconuts and bamboo as fundamental research in CNT nanotechnology. Single-walled CNTs (SWNTs) with a few carboxylic acid groups (3–5%) and pristine multi-walled CNTs (MWNTs) were mixed in each detergent solution and sonicated with a bath-type sonicator. The prepared suspensions were characterized using absorbance spectroscopy, scanning electron microscopy, and Raman spectroscopy. Among the eight combinations of CNTs and detergents (two types of CNTs and four detergents, including sodium dodecyl sulfate (SDS) as the standard), SWNTs/MWNTs were well dispersed in all combinations except the combination of the MWNTs and the bamboo detergent. The stability of the suspensions prepared with coconut detergents was better than that prepared with SDS. Because the efficiency of the bamboo detergents against the MWNTs differed significantly from that against the SWNTs, the natural detergent might be useful for separating CNTs. Our results revealed that the use of the “green” detergents had the advantage of dispersing CNTs as well as SDS.

## 1. Introduction

Carbon nanotubes (CNTs) are promising nanomaterials because of their extraordinary mechanical, electrical, and optical properties [1,2,3,4]. To apply CNTs to various applications such as nanodevices and nanobiodevices, preparing suspensions of CNTs is one of the primary techniques [5,6,7,8]. Dispersed forms of CNTs are required to maximize the specific physicochemical properties of CNTs, although synthesized CNTs generally form bundles [9,10,11,12,13].

Even when CNT films, not CNT suspensions, are the outputs of some studies or applications, CNTs are dispersed once before preparing the CNT films [14,15,16,17]. Furthermore, for biological applications, the preparation of aqueous dispersions of CNTs has been an important research topic. Although most biological applications such as drug delivery and nanobiosensors using CNTs are realized in aqueous solutions, CNTs are insoluble in water; therefore, CNTs settle as aggregates in aqueous solutions [18,19,20]. Dispersing CNTs is necessary for biological applications.

Various methods for “wrapping” CNTs using water-soluble organic molecules have been proposed to prepare stable CNT aqueous suspensions. The most popular molecules for wrapping CNT surfaces are surfactants such as sodium dodecyl sulfate (SDS), sodium cholate (SC), and sodium dodecylbenzene sulfonate (SDBS) [21,22,23,24,25,26]. The mixture of CNT powders and surfactant aqueous solutions are sonicated by bath-type or probe-type sonicators, and surfactant molecules wrap the CNT surfaces; therefore, water-soluble hybrids of CNTs and surfactants could be fabricated. DNA, proteins, and various polymers have also been used to wrap CNTs as well as surfactants [27,28,29]. Furthermore, comparisons of the dispersion efficiency of several different dispersants have been reported to optimize the dispersion conditions of CNTs [30,31,32,33,34,35,36,37].

In previous studies that reported hybridizations of surfactants and CNTs, synthetic surfactants such as SDS were employed, although there are various natural surfactants from plants that are more ecological than synthetic surfactants. In this study, we examined three commercially available natural detergents made from coconuts and bamboo for dispersing single-walled CNTs (SWNTs) and multi-walled CNTs (MWNTs) in order to evaluate the advantages and disadvantages of the use of “green molecules” for carbon nanotechnology.

Additionally, considering the use of CNT suspensions for general applications, we demonstrated treatments with a bath-type sonicator rather than a probe-type sonicator. Although both bath-type and probe-type instruments are widely used for preparing CNT suspensions, bath-type sonicators might be more suitable for general use.

## 2. Results

We employed two types of CNTs and three commercially available natural detergents. One of the CNTs was SWNTs, which had 1.0 to 3.0 atomic % of carboxylic acid group based on the technical information provided by the manufacturer. It is expected that SWNTs can be used in various biological applications by derivatizing them with various functional groups. The other is intrinsic MWNTs. It is cheaper than SWNTs and functionalized MWNTs; thus, it can be widely applied in industrial applications. For detergents, SDS and three commercially available natural detergents were used. SDS is one of the most popular synthetic detergents; thus, the availability of natural detergents can be compared with SDS. The three natural detergents are widely sold in traditional and internet markets for general use. Two of these were based on coconuts; therefore, alkyl ether sulfate ester salt and fatty acid alkanolamide were the major components. The two coconut-based detergents from different manufacturers will be described as Coco1 and Coco2 in the following sentences. These are among the most popular “green molecules.” Bamboo detergents produced from bamboo coal and bamboo ash were also suitable “green molecules” for CNT solubilization.

The preparation procedure for the CNT suspension was as follows: The SWNT or MWNT powders were mixed with 0.5% detergent aqueous solutions. Because the compositions of the commercial detergents are not fully disclosed, the surfactant concentrations of the purchased products were defined as 100%. The detergent concentrations of Coco1 and Coco2 were 15% and 16%, respectively. Thus, the final surfactant concentrations were estimated to be approximately 0.075 or 0.08%. In the case of SDS, the final SDS concentration in the sample was 0.5%. Mixtures of SWNT/MWNT powders and detergents were sonicated using a bath-type sonicator in a glass vial for one h on ice. The sonication was stopped every 15 min to add ice. During sonication, eight samples (combinations of two types of CNTs and four detergents) were set at the center of the water bath, and the configuration of the samples was randomly changed. Among the seven independent repeats, three data points were used for analysis.

**Figure 1:**
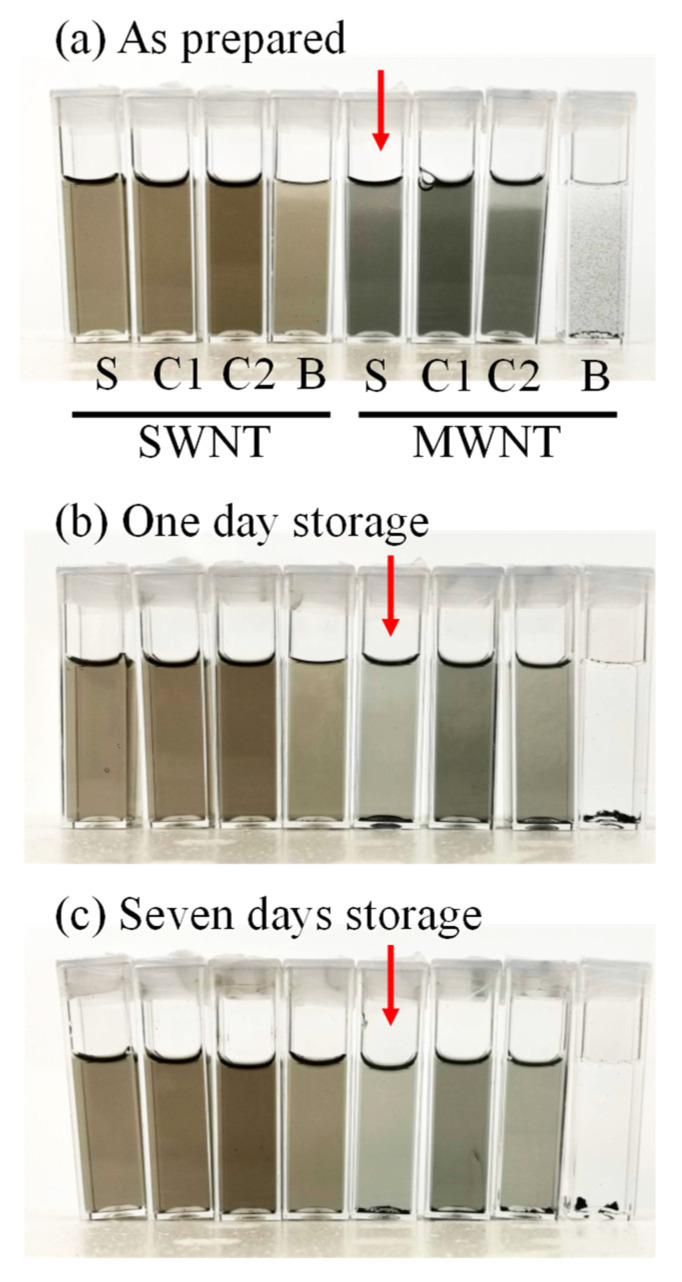
Photographs of SWNT and MWNT suspensions in the optical cuvettes. The prepared suspensions were diluted 30 times (final concentration: 0.017 mg/mL CNTs, 0.017% detergents), and 500 μL of the diluted suspensions were sealed in an optical cuvette. (**a**) As-prepared (**b**) 1-day storage (**c**) 7-day storage S: SDS, C1: Coconut1, C2: Coconut2, B: Bamboo.

Figure 1 shows the typical photographs of the prepared CNT suspensions. After sonication, each suspension was diluted 30 times in a cuvette. Centrifugation and removal of the detergents were not performed. Photographs in Figure 1a were taken as soon as possible after sample preparation. CNTs were well dispersed with natural detergents, as well as with the standard detergent SDS. As we described, the actual surfactant concentrations of the natural detergents were likely lower than those of SDS. MWNTs, with bamboo as the only exception. In the combination, many visible aggregates were found, which were settled even when capturing pictures. The absorbance spectra were stable in the other combinations during the measurements. We will skip quantitative discussions with these pictures because sometimes fluctuating results appeared in the experiments. CNT powders are very light because of their large surface areas, resulting in rather higher fluctuations of the data than the usual experiments. For this reason, we independently repeated the same experiments seven times and analyzed the middle three data among the seven data sets.

The samples were re-observed after 1 day of storage (Figure 1b) and 7 days of storage (Figure 1c) to evaluate the stability of the suspensions. The cuvettes were stored at room temperature without shaking. In the aging experiments, the prepared suspensions were diluted 30 times with 20 mM phosphate buffer solution without detergents. Thus, the final concentration of each detergent in the stored samples was 0.017%. Interestingly, MWNTs dispersed with SDS were settled even after 1 day of storage, although SDS is a popular compound for dispersing CNTs (see the arrows in Figure 1). With coconut-based Coco1 and Coco2, the concentrations of suspended CNTs slightly decreased. We believe that this is an advantage of using natural detergents.

In the case of bamboo detergent, most of the MWNTs settled after 1 day of storage. In contrast, the SWNTs with bamboo were stable. It should not be suggested that bamboo detergents can distinguish SWNTs and MWNTs. Although we attempted to suspend pristine SWNTs with the four detergents as preliminary experiments, the SWNTs were less dispersed (data not shown). As one possible speculation, the bamboo detergent can disperse CNTs with a few carboxyl groups (1.0 to 3.5%) due to technical data from the manufacturer). Although further experiments are necessary to conclude, the bamboo detergent might be useful for separating specific CNTs from a mixture of different types of CNTs. The separation of different types of CNTs has been an important research topic because the extraordinary physicochemical properties of CNTs are illuminated with purified CNTs [38,39,40,41].

The suspensions were examined using UV-vis absorbance spectroscopy to obtain quantitative information. The absorbance spectra obtained are shown in Figure S1. Each line shows the average of the middle three data points among the seven repeats.

**Figure 2:**
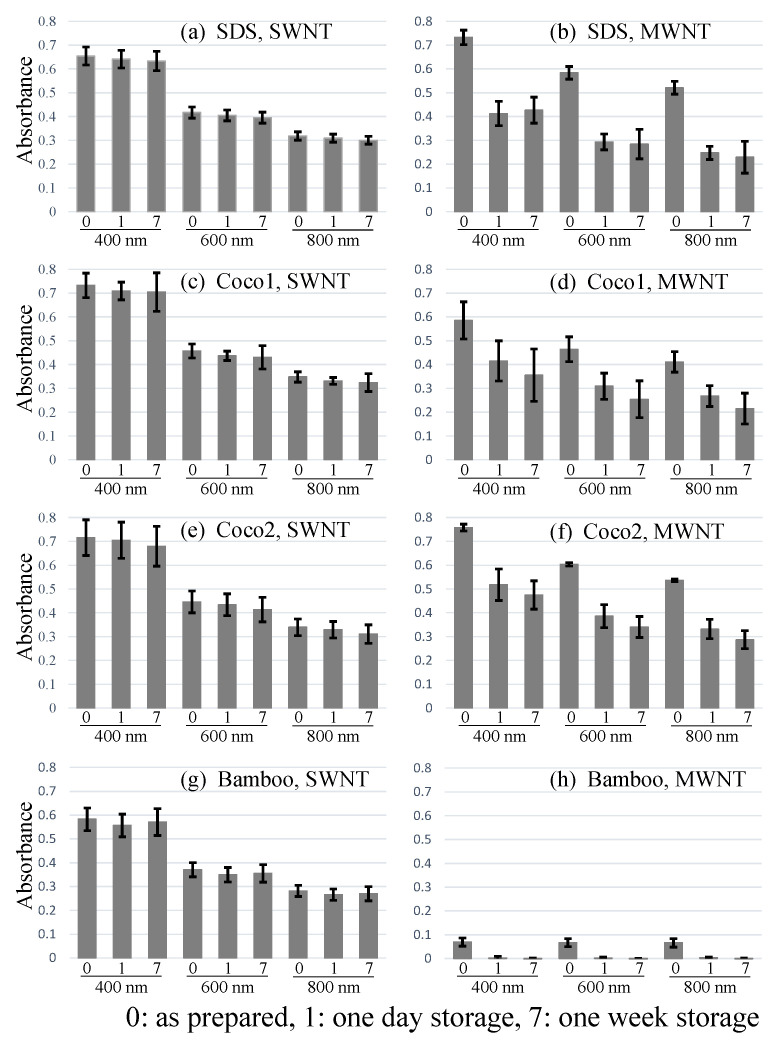
Histograms of absorbance of CNT suspensions at 400, 600, and 800 nm. (**a**) SWNTs with SDS, (**b**) MWNTs with SDS, (**c**) SWNTs with Coco1, (**d**) MWNTs with Coco1, (**e**) SWNTs Coco2, (**f**) MWNTs with Coco2, (**g**) SWNTs with Bamboo, (**h**) MWNTs with Bamboo. 0: As prepared, 1: 1-day storage, 7: 7-days storage.

Figure 2 shows histograms of absorbance values at 400, 600 and 800 nm obtained from the UV-vis spectra. The absorbance values of SWNTs with SDS, Coco1, and Coco2 were almost similar at 400, 600, and 800 nm. Although the average value with SDS was slightly smaller than that with Coco1 and Coco2, it was in the range of fluctuation in our experimental accuracy. The absorbance of bamboo was almost 15% lower than that with SDS, Coco1, and Coco2. As we already pointed out, moderate dispersant performance might be useful for the separation of CNTs. In the case of SWNTs, absorbance values slightly decreased after 1 or 7 days of storage.

In the case of MWNTs, the absorbance with bamboo was only 10% of that with SDS. The absorbance value became almost zero after 1 day of storage. This is in complete agreement with the visualized results shown in Figure 1. The MWNTs with SDS and Coco2 exhibited similar absorbance values. The absorbance of Coco1 was almost 20% lower than that of SDS and Coco1. This suggests that each detergent had some specificity against CNTs, although the mechanism is not clear at this moment.

This is a significant phenomenon. When the MWNT samples with SDS were stored, the absorbance decreased by nearly 50% after 1 day of storage. After 7 days of storage, the absorbance values no longer changed. In contrast, the absorbance values of MWNTs with Coco1 and Coco2 decreased by 30% to 40% after 1 day of storage and then decreased by an additional 10% after 7 days of storage. This suggests that Coco1 and Coco2 provided a more stable dispersion over SDS.

**Figure 3:**
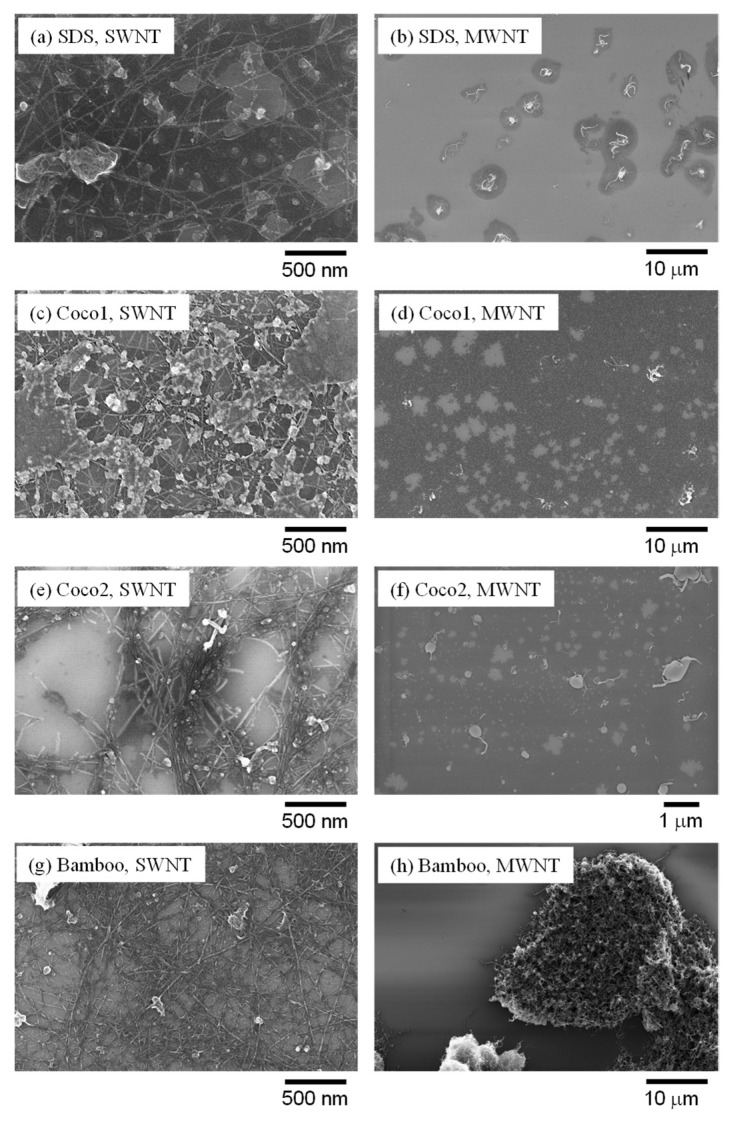
SEM of absorbance of CNT suspensions at 400, 600 and 800 nm. (**a**) SWNTs with SDS, (**b**) MWNTs with SDS, (**c**) SWNTs with Coco1, (**d**) MWNTs with Coco1, (**e**) SWNTs Coco2, (**f**) MWNTs with Coco2, (**g**) SWNTs with Bamboo, (**h**) MWNTs with Bamboo.

The prepared suspensions were characterized using scanning electron microscopy (SEM) and Raman spectroscopy. Figure 3 shows the typical SEM images of the eight types of suspensions. SWNTs were well dispersed, although dried detergents were also observed, like films. Although the absorbance of SWNTs with bamboo was smaller than those with other detergents, the observed SWNTs were well dispersed (Figure 3g).

MWNTs with SDS, Coco1, and Coco2 were also well dispersed. Only MWNTs with bamboo detergent revealed large aggregates (Figure 3h). Dispersed MWNTs were not observed in the sample. This observation corresponded with the results of visual observations (Figure 1) and absorbance spectra (Figure S1 and Figure 2h). The results clearly indicate that the bamboo detergent distinguished the SWNTs and MWNTs. It might be beneficial to use detergents to separate the CNTs.

SWNTs and MWNTs sonicated in a phosphate buffer solution without any detergents were also observed by SEM. In both cases, only aggregates were observed (data not shown). Further analysis of the structures of dispersed CNTs with higher resolution is needed.

Raman spectroscopy (Figures S2 and S3) confirmed that sonication and other processes did not affect the structures of the CNTs. There were no differences in the G/D ratios even after sonication. The peak wavenumbers of the SWNT powder, SWNT/SDS, SWNT/Coco1, SWNT/Coco2, SWNT/bamboo, MWNT powder, MWNT/SDS, MWNT/Coco1, MWNT/Coco2, and MWNT/bamboo were 1350, 1342, 1338, 1342, 1342, 1350, 1353, 1350, 1350, and 1353 cm^−1^, respectively. Husanu et al. reported differences in the Raman wavenumbers of SWNTs in G and radial breathing mode (RBM) peaks. [42] They suggested that the wavenumbers of the G and RBM bands of isolated SWNTs were slightly smaller than those of bundled SWNTs. Based on previous studies, the SWNT/Coco1 could be isolated in our data. No wavenumber shifts were observed in the RBM peaks. Additionally, there was a small peak at 1430 cm^−1^ that we cannot sufficiently explain.

**Figure 4:**
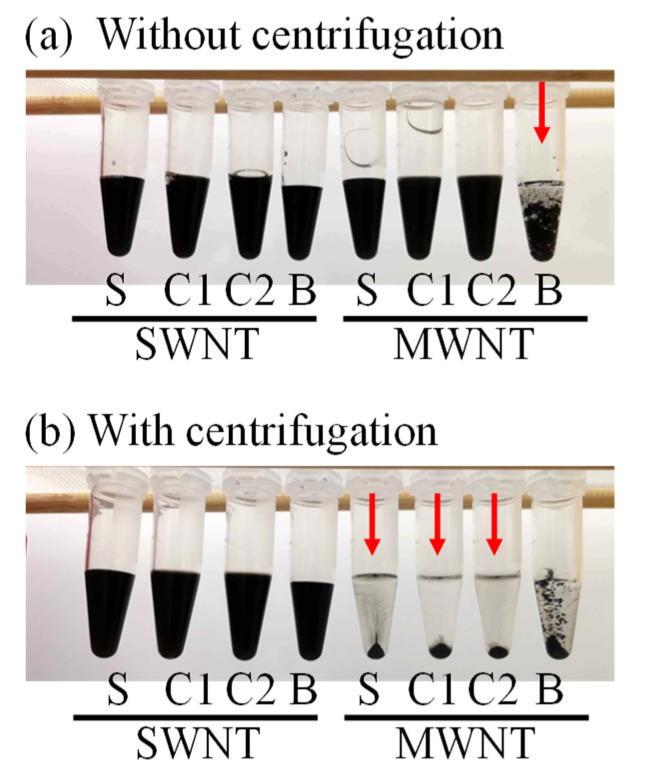
Photographs of prepared CNT suspensions. (**a**) Without centrifugation. (**b**) With centrifugation. S: SDS, C1: Coconut1, C2: Coconut2, B: Bamboo.

Finally, we demonstrated the centrifugation of the samples 7 days after sonication. Each suspension (500 μL) was transferred from a vial to a plastic tube and centrifuged at 15,000 rpm (21,482× *g*) for 1 h. As a result, most of the MWNTs precipitated, and most of the SWNTs were not precipitated (Figure 4). This suggests that the stability of the suspension was quite different between the SWNTs and MWNTs.

## 3. Methods

SWNTs (P3-SWNT, Carbon Solutions, Inc., Riverside, CA, USA) [43] and MWNTs (C2158, Tokyo Chemical Industry Co., Ltd., Tokyo, Japan) were used as received. Sodium dodecyl sulfate (SDS, 192-08672; Wako Pure Chemical Corporation, Osaka, Japan). Coconut-based YASHINOMI Detergent (Saraya Co. Ltd., Osaka, Japan) including 16% surfactants (alkyl ether sulfate ester salt and fatty acid alkanolamide) and coconut-based COCONUTS detergent (Blue Sea International Co., Saitama, Japan), including 15% surfactants (alkyl ether sulfate ester salt and fatty acid alkanolamide) were used as received. These were made from the endosperm of cocos nucifera. Bamboo-based BAMBOO CLEAR detergent (Upside Co., Osaka, Japan), including bamboo coal and bamboo ash (concentrations are not available to the public from the manufacturer) were used as received. In this manuscript, these detergents are described as SDS, Coco1, Coco2, and Bamboo. These are also abbreviated as S, C1, C2, and B in the figures, respectively.

SWNT (0.5 mg) was mixed with 1 mL of 20 mM phosphate buffer solution in a glass bottle. Detergents (final concentration: 0.5%) were added to the mixture, except for the control samples. The mixture was sonicated in a bath-type sonicator (W-113 MKII, 31 kHz, 110 W, HONDA ELECTRONICS CO. LTD., Aichi, Japan) for 1 h (15 min × 4 times). Ice was floated in the water bath to avoid an increase in temperature. The ice was replenished every 15 min. The samples were shaken every 15 min during sonication to obtain a uniform dispersion.

A UV-Vis/NIR spectrophotometer (V-630, JASCO Corporation, Tokyo, Japan) was used to obtain the absorbance spectra (200–1100 nm). The samples (100 μL) were diluted with 2900 μL of 20 mM phosphate buffer solution in plastic cuvettes. The absorbance spectra of the same samples were measured three times (as soon as possible, 1 day after, and one week after). Photographs of the cuvettes with diluted samples were also obtained three times (as soon as possible, 1 day after, and one week after). These experiments were repeated five times independently.

Raman spectra were obtained using a microscopic Raman spectrometer (RAMANtouch i VIS-NIR, Nanophoton Corporation, Osaka, Japan). Fifty μ microliters of the samples were dropped onto a slide glass without dilution and then dried in air. Raman spectra were measured using a 20× objective lens in air. The excitation wavelength was 532 nm.

Scanning electron microscopy (SEM, SUPR40, Carl Zeiss AG, Oberkochen, Germany) was used to confirm the degree of CNT dispersion. SEM samples were prepared using a spin coater (MS-B100, Mikasa). The CNT solutions were spin-coated onto thermally oxidized Si wafers (Electronics and Materials Co. Ltd., Hyogo, Japan) at a rotation speed of 1500 rpm for 60 s, followed by drying in air at room temperature.

Samples were centrifuged after 7 days of storage to examine the stability of the suspensions. For the experiments, 500 μL of the suspension was centrifuged at 1500 rpm (21,482× *g*) for 1 h without dilution.

## 4. Conclusions

We described that natural detergents based on coconuts and bamboo are useful for dispersing CNTs. The coconut detergents were powerful, although the bamboo detergent showed different results. Our results suggest that natural detergents can be applied not only to disperse CNTs but also to separate CNTs. As a potential application, the biodegradability of natural detergents can be utilized. For example, after preparing CNT films with natural detergents, detergents can be degraded. Our work illuminated the possibility of “green molecules” for CNT nanotechnology.

## Data Availability

Data are contained within the article and Supplementary Materials.

## Supplementary Materials

The following are available online, Figures S1–S3.

## Sample Availability

Samples are not available from the authors.
