# Supplementary material for: Dispersion of Carbon Nanotubes with “Green” Detergents
Source: Molecules. 2021 May 14;26(10):2908. doi: 10.3390/molecules26102908 (PMC8153609; doi:10.3390/molecules26102908)
Supplement: Supplementary file 1 [file molecules-26-02908-s001.zip › molecules-1182412-supplementary.pdf]

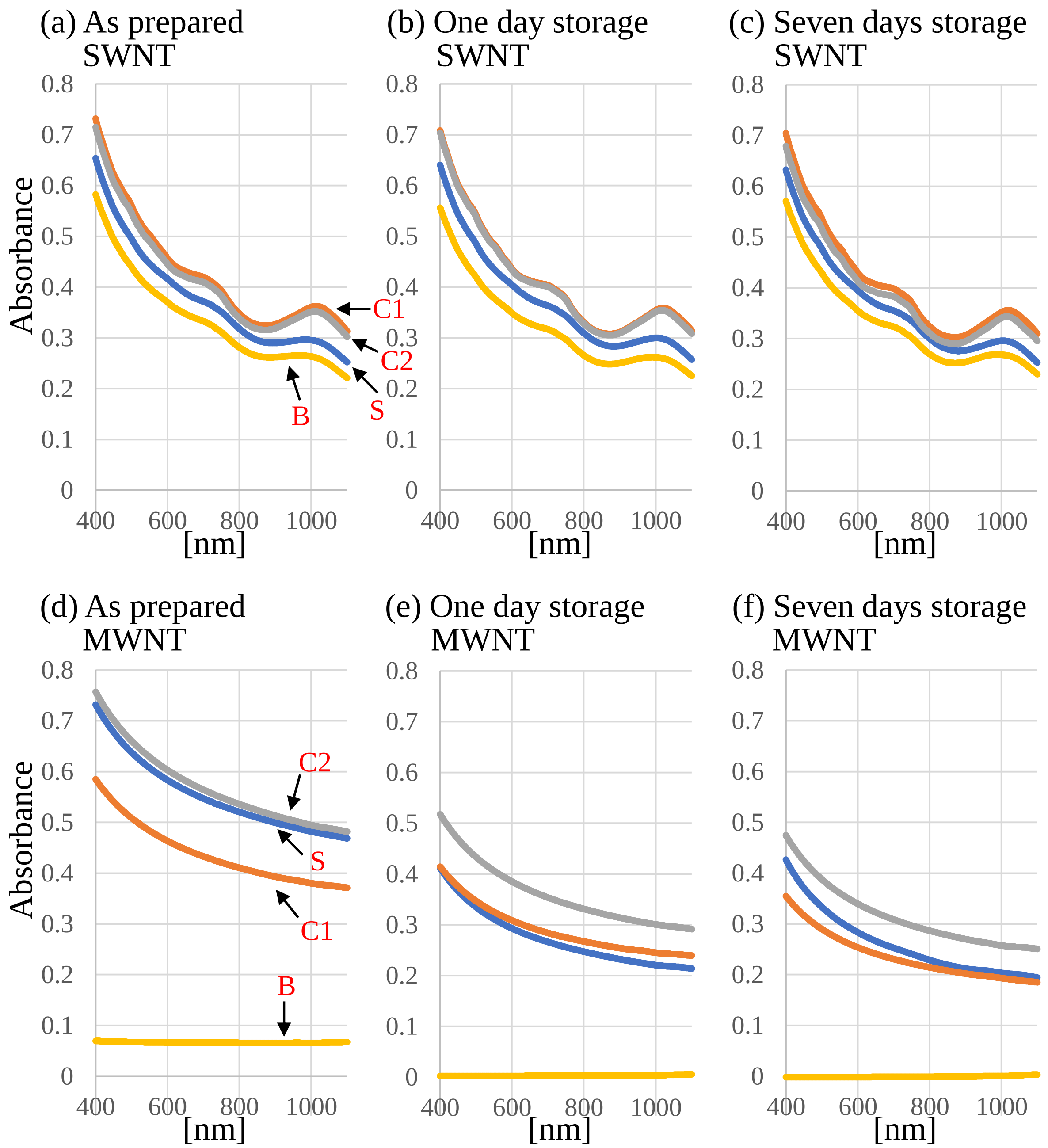

Figure S1. UV-vis absorbance spectra of prepared CNT suspensions. (a) As prepared, SWNTs. (b) 1 day storage, SWNTs. (c) 7 days storage, SWNTs. (d) As prepared, MWNTs, (b) 1 day storage, MWNTs, (c) 7 days storage.

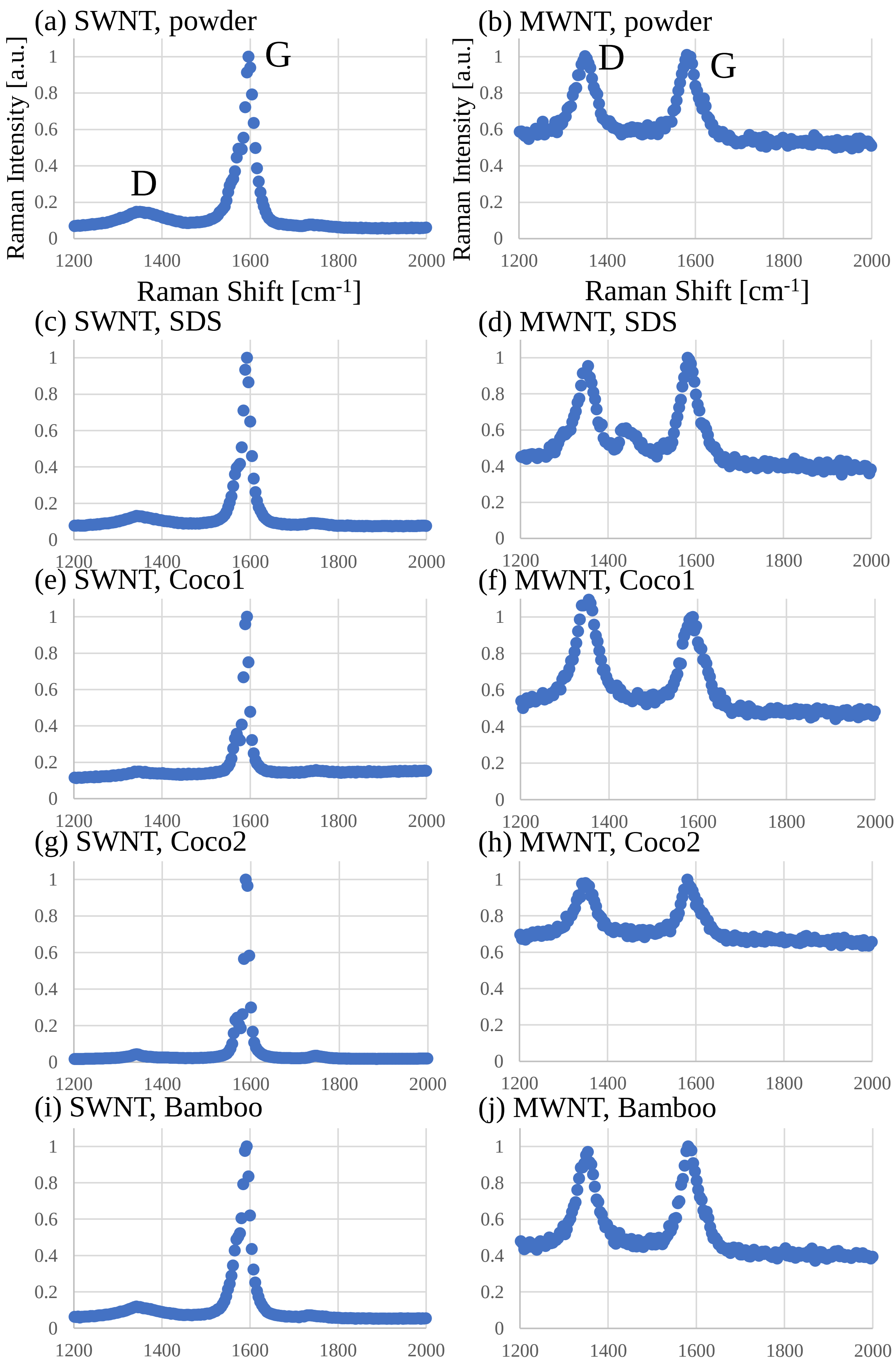

Figure S2. Raman spectroscopy of prepared CNT suspensions (G/D region). (a) SWNT powder, (b) MWNT powder, (c) SWNTs with SDS, (d) MWNTs with SDS, (e) SWNTs with Coco1, (f) MWNTs with Coco1, (g) SWNTs with Coco2, (h) MWNTs with Coco2, (i) SWNTs with Bamboo, (j) MWNTs with bamboo.

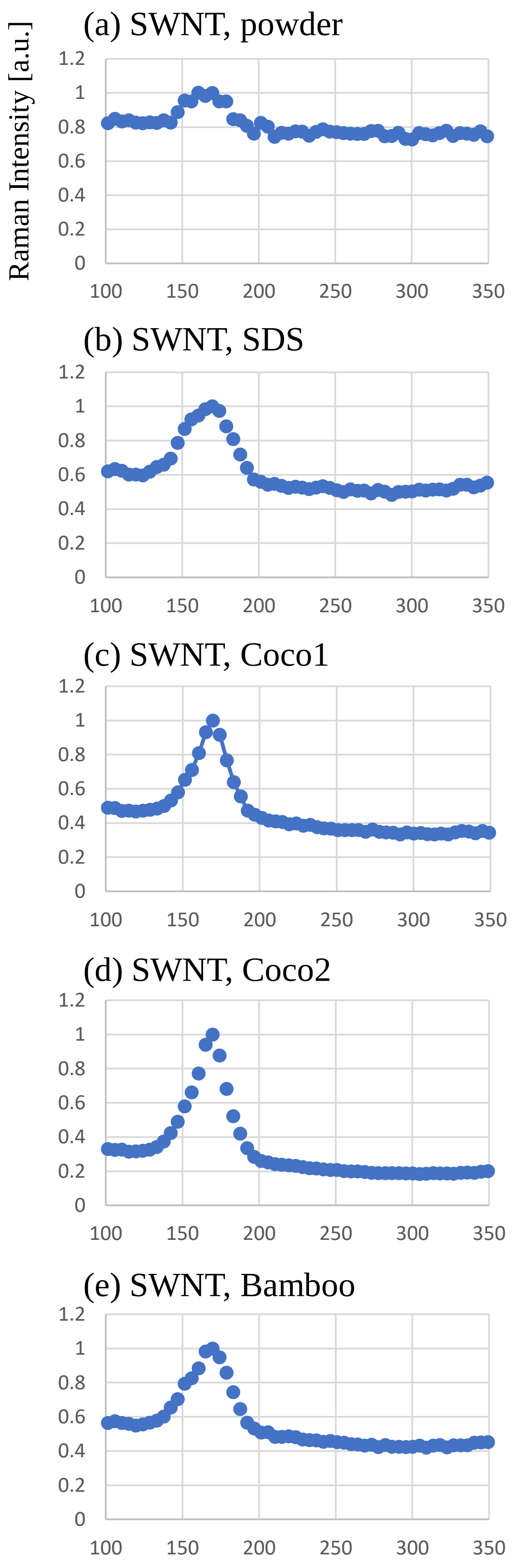

Figure S3. Raman spectroscopy of prepared CNT suspensions (RBM region). (a) SWNT powder, (b) SWNTs with SDS, (c) SWNTs with Coco1, (d) SWNTs with Coco2, (e) SWNTs with Bamboo.
